# Evaluating the effects of empagliflozin in preventing myocardial injury in patients undergoing percutaneous coronary intervention: A double-blind, randomized clinical trial

**DOI:** 10.34172/jcvtr.33103

**Published:** 2024-06-25

**Authors:** Hossein Behzad, Sina Mashayekhi, Hila Asham, Parvin Sarbakhsh, Taher Entezari-Maleki

**Affiliations:** ^1^Department of Clinical Pharmacy, Faculty of Pharmacy, Tabriz University of Medical Sciences, Tabriz, Iran; ^2^Cardiovascular Research Center, Tabriz University of Medical Sciences, Tabriz, Iran; ^3^Department Research Center, Faculty of Public Health, Tabriz University of Medical Sciences, Tabriz, Iran

**Keywords:** Empagliflozin, Percutaneous coronary intervention, Periprocedural myocardial injury, cTnI, hs-CRP

## Abstract

**Introduction:**

Percutaneous Coronary Intervention (PCI) is a fundamental procedure for coronary artery disease management, yet the risk of adverse events such periprocedural myocardial injury (PMI) persists. This double-blind, randomized clinical trial aims to assess the efficacy of empagliflozin in preventing myocardial injury during PCI procedure.

**Methods:**

A total of 90 patients were randomly assigned to two groups A and B; Group A as the intervention group received empagliflozin 25 mg 24 hours before and empagliflozin 10 mg 1-2 hours before coronary intervention and group Bas the control group received placebo at similar intervals. The primary outcome involved comparing baseline, 8-hour, and 24-hour cTnI and baseline and 24-hour hs-CRP levels after PCI in both groups to measure the incidence of periprocedural myocardial injury (PMI) and anti-inflammatory effects of empagliflozin.

**Results:**

Baseline cTnI levels with *P*=0.955, 8 hours after PCI with *P*=0.469, and 24 hours after the intervention with *P*=0.980 were not statistically different in the two groups. Baseline levels of hs-CRP in both intervention and control groups were not statistically significantly different (*P*=0.982). Also, there was no statistically significant difference in hs-CRP levels 24 hours after PCI in two groups (*P*=0.198). Finally, the results showed that MACEs did not occur in any of the groups.

**Conclusion:**

The results of this trial could not express the advantages of acute pretreatment with empagliflozin in preventing PCI-related myocardial injury.

## Introduction

 Cardiovascular disease (CVD) remains a leading cause of morbidity and mortality worldwide, with myocardial infarction (MI) representing a significant contributor to this global burden.^1^ Percutaneous coronary intervention (PCI), a widely employed revascularization procedure, has dramatically improved outcomes in patients with coronary artery disease (CAD).^2^ However, despite its effectiveness, PCI can provoke myocardial damage, termed periprocedural myocardial injury (PMI), which may ultimately lead to adverse clinical outcomes. PMI has been linked to an increased risk of future major cardiovascular events and can contribute to a poorer prognosis for patients.^3,4^

 Empagliflozin, a sodium-glucose cotransporter 2 inhibitors (SGLT2-I) initially developed for managing type 2 diabetes mellitus (T2DM), has recently garnered significant attention for its potential cardiovascular benefits beyond glycemic control.^5^ Clinical trials such as the EMPA-REG outcome trial have remarkably reduced cardiovascular mortality, heart failure hospitalization, and adverse cardiac events in patients with T2DM receiving empagliflozin. These findings have sparked interest in exploring empagliflozin’s utility in the PCI context to mitigate PMI and improve clinical outcomes.^6,7^

 The pathophysiology of PMI is multifaceted, involving ischemia-reperfusion injury, inflammation, oxidative stress, and endothelial dysfunction.^8^ Empagliflozin has demonstrated pleiotropic effects that extend beyond glucose lowering. It exerts its cardio protective effects by modulating various mechanisms, including natriuresis, diuresis, and reductions in blood pressure, arterial stiffness, and intravascular volume.^9^ Additionally, empagliflozin has been shown to enhance myocardial energetics, reduce myocardial fibrosis, and improve endothelial function, all of which may play a role in protecting against PMI.^10^

 Despite these promising preclinical and clinical findings, there remains a paucity of research investigating the specific impact of empagliflozin on PMI in patients undergoing PCI. Given the potential benefits of empagliflozin on cardiac function and its favorable safety profile, it is imperative to explore whether its administration before or after PCI can effectively reduce PMI and improve post-procedural outcomes.^11,12^ Therefore, the present study aims to evaluate the effects of empagliflozin in preventing myocardial injury in patients undergoing PCI.

## Materials and Methods

 The present study was conducted prospectively, randomly, and in a double-blind clinical trial at the Shahid Madani Heart Center, the largest cardiovascular center in northwestern Iran, with a sample size of 90 patients (45 individuals in each group).

###  Inclusion and exclusion criteria

 The inclusion criteria for this study included ischemic heart patients aged 18 to 80 years old who were candidates for elective PCI, including angioplasty with balloon and stent placement. Also, the exclusion criteria for this study included patients who met the following criteria: elevated levels beyond the reference range of cardiac troponin I(cTnI) and hs-CRP before PCI, acute myocardial infarction or a history of myocardial infarction, open-heart surgery within the last 3 months, unsuccessful PCI, patients with kidney dysfunction (eGFR < 30 ml/min), patients undergoing dialysis, patients in cardiogenic shock, severe infections, cancer, liver failure (Child Pugh score > 6), uncontrolled autoimmune and inflammatory diseases, pregnancy and lactation, a history of serious hypersensitivity reactions to empagliflozin or any component of the formulation, type 1 diabetes mellitus, diabetic ketoacidosis, and patients who did not provide informed consent.

###  Trial procedures 

 Participants were divided into two groups, A and B, using GraphPad Prism software and a 1:1 randomization method. Group A served as the intervention group and received empagliflozin 25 mg 24 hours before and empagliflozin 10 mg 1-2 hours before coronary intervention. Group B, on the other hand, served as the control group and received a placebo 24 hours before and 1-2 hours before coronary intervention.

###  Selection of patient

**Figure 1:**
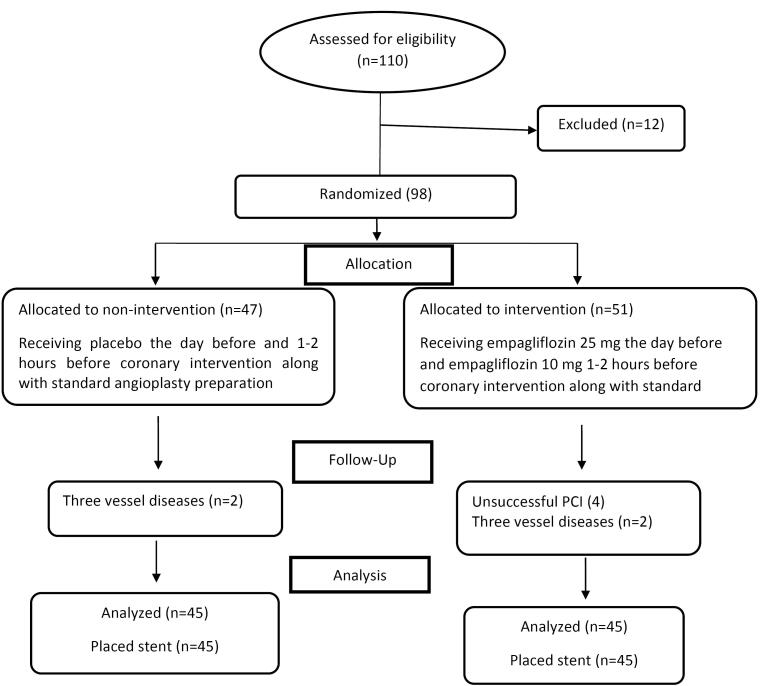
Patients’ selection and randomization

 The total number of patients included in the evaluation was 110, five patients due to high baseline levels of cTnI, four patients due to a history of infarction or open-heart surgery in the last three months, two patients due to renal failure, and one patient due to type 1 diabetes mellitus, were excluded from the study. After the intervention and in the follow-up phase, the data of eight patients (four patients due to unsuccessful PCI and four patients with simultaneous involvement of three vessels) were excluded. Finally, 90 patients including 45 patients in the intervention group and 45 patients in the control group were included in the analysis. The study process is shown in Figure 1.

 The study was conducted as a double-blind trial, and participants and the researcher were unaware of the group assignments, which were coded as A and B. All participating patients followed standard preparations before angioplasty, including the consumption of aspirin 325 mg, clopidogrel 300 mg (4 tablets of 75 mg each), and intravenous heparin (with a goal of achieving a clotting time of 250-350 seconds) uniformly. Additionally, both groups received 100 ± 20 cc of the contrast agent iodixanol during the PCI procedure. To prevent contrast-induced nephropathy, hydration with isotonic saline (1ml/kg/h) was administered from 6-12 hours before the procedure until 6-12 hours after. All participants underwent PCI by the same standardized international team that was unaware of the individual group assignments.

 During a one-month follow-up period, all patients were assessed for major adverse cardiac events (MACEs), including death, Q-wave myocardial infarction, revascularization of the target vessel, and ischemic stroke, as secondary outcome.

 Patient demographic information, including gender, age, weight, height, BMI, medication history, medical history, laboratory data, and a positive family history of cardiovascular diseases, were recorded on data collection forms.

###  Blood sampling

 Blood sampling was conducted according to the Fourth Universal Definition of Myocardial Infarction (UDMI). Baseline levels of the cardiac biomarker cTnI were measured before PCI and before empagliflozin administration (25 mg and placebo). Measurements were also taken at 8 and 24 hours after PCI. Additionally, baseline hs-CRP levels and 24 hours post-intervention were measured and recorded to evaluate inflammation.

 The level of serum CRP was measured using hs-CRP immunoassay test kits (Aptec Diagnostics, 9100 Sint-Niklaas, Belgium; 9100 Sint-Niklaas, Belgium). The measurement range is 0 to 14 mg/dL with a detection limit of 0.013 mg/dL. The risk of cardiovascular diseases will be determined based on the kits reference values: less than 0.10 mg/dL as low-risk, 0.10 to 0.30 mg/dL as intermediate-risk, and greater than 0.30 mg/dL as high-risk. Serum troponin level was determined using an ELISA sandwich method (Monobind Inc, USA) with a detection limit of 0.1 ng/ml and upper limit normal of 1.2 ng/ml.

###  Study Outcomes

 The primary outcome involved comparing baseline, 8-hour, and 24-hour cTnI levels after PCI in both groups to assess the occurrence of PMI. Furthermore, a comparison of baseline and 24-hour hs-CRP levels in the groups was conducted to investigate the anti-inflammatory effects of empagliflozin. The secondary outcome was the incidence of major cardiac events (death, Q-wave myocardial infarction, revascularization of the target vessel, and ischemic stroke) during a one-month follow-up after PCI.

###  Sample size and post-hoc power calculation

 Due to the lack of similar studies, the present study’s sample size determined as a pilot subject of 45 individuals in two groups based. The post-hoc power calculation was also performed with the sample size of 90, two equal groups, three times of measurements, and α = 0.05 using G*Power 3.1.9.2. The power (1−βerror) for cTnI test with partial η 2 of 0.057 and calculated effect size (F) of 0.245 was calculated 0.80.

###  Statistical analysis

 Data were analyzed in SPSS 26 software. The normal distribution of the data was evaluated with the Kolmogorov-Smirnov test, and quantitative variables were evaluated with the Chi-square test or Fisher exact test. Repeated measure ANOVA was used for within-between comparisons by Bonferroni adjustment. Continues variable comparison between two groups was performed using independent t-test method or Mann-Whitney U if appropriated. P-values of 0.05 or below were considered significant for all outcomes.

## Results

 The intervention group included 25 women (55.5%) and 20 men (44.5%), and the control group included 19 women (42.2%) and 26 men (57.8%). The mean ± SD for the age of the patients in the intervention and control groups was 59.2 ± 9 and 59.4 ± 9.3, respectively, which indicates the prevalence of ischemic heart diseases in the elderly. Most of the targeted stent vessels were left anterior descending artery (LAD), with 49% and 44.4% in intervention and control groups, respectively. About one-third of patients were one-vessel disease [n = 17 (37.7%) vs. n = 15 (33.3%)] in the intervention and the control groups respectively (*P* = 0.66). The mean ± SD number of stents per patient was 1.49 ± 0.7 in the intervention group and was 1.35 ± 0.6 in the control group. All patients received drug eluted stents. Basic demographic and clinical information of the patients has no significant difference (*P* < 0.05) (Table 1).

**Table 1:** Demographic and clinical information of the subjects in both groups

**Variable**	**Intervention (n=45)**	**control (n=45)**	***p*** **-value**
Age (years)	59.2 ± 9	59.4 ± 9.3	0.93
Gender Female Man	25 (55.5%) 20 (44.5%)	19 (42.2%) 26 (57.8%)	0.2
Weight (kg)	76.53 ± 13.9	79.57 ± 9.6	0.23
Height (m)	1.66 ± 0.06	1.66 ± 0.06	0.52
BMI(kg/m2)	27.65 ± 4.4	28.59 ± 3.3	0.264
Smoking	17 (37.7%)	18 (40%)	0.829
DM	6 (13.3%)	10 (22.2%)	0.27
HTN	33 (73.3%)	34 (75.5%)	0.8
Dyslipidemia	45 (100%)	45 (100%)	1
History of other diseases	14 (31.1%)	11 (24.4%)	0.48
Family history of heart disease	17 (37.7%)	17 (37.7%)	1
History of open-heart surgery	0	0	1
PCI history	0	0	1
Use of cardiovascular drugs	43 (95.5%)	45 (100%)	0.494
Use of antidiabetic drugs	6 (13.3%)	10 (22.2%)	0.27
Use of dyslipidemia drugs	38 (84.4%)	41 (91.1%)	0.334
Use of other medication	32 (71.1%)	31 (68.8%)	0.81
LAD	22 (49%)	20 (44.4%)	0.673
RCA	11 (24.4%)	13 (28.8%)	0.634
LCX	8 (17.7%)	10 (22.2%)	0.598
OM	7(15.5%)	6 (13.3%)	0.764
Other vessels	8 (17.7%)	7 (15.5%)	0.777
One-vessel disease	17 (37.7%)	15 (33.3%)	0.660
Two-vessel disease	12 (26.6%)	10 (22.2%)	0.231
Three-vessel disease	5 (11.1%)	7 (15.5%)	0.535
Total stents diameter (mm), mean ± SD	2.8 ± 0.47	2.9 ± 0.5	0.210
Total stents length (mm), mean ± SD	21 ± 6.4	19.5 ± 5.3	0.205
Number of stents per patient, mean ± SD	1.49 ± 0.7	1.35 ± 0.6	0.278

Data has been presented as N (%), mean ± SD or median (IQR) which is appropriate. BMI, body mass index; DM, diabetes mellitus; HTN, hypertension; LAD, left anterior descending artery; LCX, left circumflex artery; OM, obtuse marginal artery; RCA, right coronary artery

**Figure 2:**
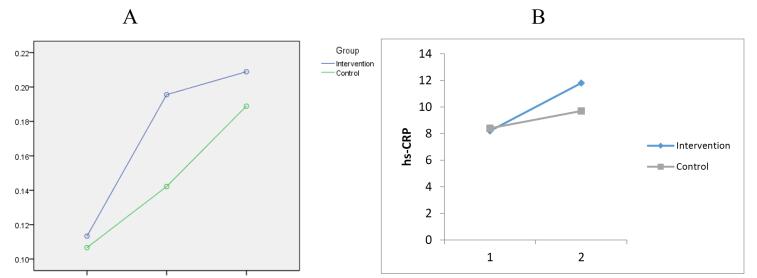
A. changes in troponin I after PCI at baseline, 8 and 24 h. B. changes in hs-CRP after PCI at baseline and 24 h

 Baseline cTnI levels with *P* = 0.955, 8 hours after PCI with *P* = 0.469, and 24 hours after the intervention with *P* = 0.980 were not statistically different in the two groups (Table 2), (Figure 2).

**Table 2:** Mean Troponin I Level at Baseline and 8 and 24 Hours After PCI

**Troponin I level**	**Intervention(n=45)**	**Control(n=45)**	***P*** **-value**
Baseline	0.11 ± 0.05	0.1 ± 0.02	0.955
8 hours after PCI	0.19 ± 0.39	0.14 ± 0.07	0.469
24 hours after PCI	0.2 ± 0.26	0.18 ± 0.26	0.980

 Baseline levels of hs-CRP in both intervention and control groups were not statistically significantly different (*P* = 0.982). Also, there was no statistically significant difference in hs-CRP levels 24 hours after PCI in the two groups (*P* = 0.198) (Table 3), (Figure 2). Finally, the results showed that MACEs did not occur in any of the groups.

**Table 3:** Mean hs-CRP Level at Baseline and 24 Hours After PCI

**hs-CRPlevel**	**Intervention (n=45)**	**Control (n=45)**	***P*** **-value**
Baseline	8.2 ± 5.9	8.4 ± 6.3	0.982
24 hours after PCI	11.8 ± 7.1	9.7 ± 6.1	0.198

 We also did not detect any significant drug-related adverse outcome such as diabetic ketoacidosis, hypoglycemia, severe infection and severe hypotension, in both study groups.

## Discussion

 Empagliflozin has shown anti-inflammatory and anti-fibrotic effects in preclinical studies. These properties may help reduce inflammation and fibrosis in the myocardium, potentially protecting it from further damage during and after PCI.^13^

 Here, we evaluated the effects of empagliflozin in preventing myocardial damage in patients undergoing PCI. The results of the study revealed that levels of hs-CRP and troponin I in both intervention and control groups were not significantly different.

 Preventing myocardial damage during PCI is a multifaceted challenge, and a comprehensive pharmacological approach involves not only cardiovascular index drugs but also various agents from different therapeutic categories. While antiplatelet medications, statins, and vasodilators continue to be essential, researching more recent medications, like anti-inflammatory medications and antioxidants, presents promising opportunities for outcome improvement.^14,15^ Newer drugs like ticagrelor and prasugrel offer greater platelet inhibition over the traditional dual antiplatelet therapy (aspirin plus a *P*_2_*Y*_12_ receptor inhibitor), lowering the risk of thrombotic events during PCI.^16^ In high-risk PCI situations, medications such as eptifibatide and abciximab work in conjunction with antiplatelet treatment to prevent platelet aggregation.^17^ Beyond lowering cholesterol, statins like atorvastatin and rosuvastatin have pleiotropic effects. They are capable of lowering peri-procedural myocardial damage due to their anti-inflammatory and anti-thrombotic characteristics.^18^ The findings of two clinical trial studies showed that a single high dose of rosuvastatin prior to PCI reduces periprocedural myocardial injury in patients with acute coronary syndrome.^19,20^ Also, two clinical trial studies revealed that a single, high-loading dose of atorvastatin reduces the incidence of periprocedural myocardial infarction in elective PCI.^21,22^ In the study of Park et al by continuous intravenous administration of landiolol as a beta-blocker, a very significant reduction in the occurrence of periprocedural myocardial ischemia was observed.^23^

 Calcium channel blockers (CCBs) are among other categories of drugs whose intracoronary injection has been investigated in the incidence of periprocedural myocardial ischemia. Despite features such as the reduction of creatine phosphokinase (CPK) during PCI, the beneficial effects on vascular tone and coronary endothelial function are still not completely clear. In the study conducted by Arora et al on 193 patients with chronic stable angina, the intracoronary administration of nicardipine could not reduce the increase of the troponin after elective PCI.^24^

 The results of a meta-analysis study (2021) reported that the administration of pre-procedure trimetazidine may have a role in reducing periprocedural myocardial injury in patients with CAD undergoing PCI.^25^ Shah et al study (2020) showed that acute preprocedural administration of colchicine reduced the increase in interleukin-6 and hs-CRP concentrations after PCI when compared with placebo but did not lower the risk of PCI-related myocardial injury.^26^

 Aslanabadi et al (2018) conducted a randomized, controlled trial study to evaluate the effect of vitamin D in preventing cardiac injury in patients undergoing elective PCI. The results of this study were in line with the results of the current research and showed a non significant effect of vitamin D on cardiac biomarkers; however, the mean difference in CK-MB between 8 and 24 hours and the mean difference in hs-CRP were significantly in favor of the vitamin Dgroup.^27^

 The findings of a pilot randomized clinical trial (2021) did not support the potential cardio-protective benefits of allopurinol administration on decreasing myocardial injury following non-ST elevation myocardial infarction.^28^

 The effects of SGLT2-Is on primary PCI among patients with acute coronary syndrome (ACS) were evaluated through previous studies. The results of the Adel et al study (2022) showed that the empagliflozin and placebo groups were not significantly different compared to the standard treatment results in terms of incidence of death, hospitalization due to unstable angina, and coronary revascularization after treatment.^29^ Compared to our study, in this review diabetic patients were randomly assigned to receive empagliflozin (10 mg once daily) or placebo at similar doses for 6 months after primary PCI, but in our study patients in the intervention group received two doses of this drug after elective PCI. The results of another study revealed that diabetic individuals with acute myocardial infarction (AMI) who underwent primary PCI and were treated with SGLT-Is had lower CRP levels on admission compared to those in the control group, with a statistically significant difference (*P* < 0.001). This observational study also evaluated the level of CRP after 24 hours and at discharge (a median of 5 days), showing significant beneficial effects of SGLT-Is in lowering CRP levels (*P* = 0.01 for both).^30^ However, our study did not show a significant effect of empagliflozin among individuals undergoing elective PCI.

 This randomized-double-blinded clinical trial is the first of its kind evaluated the effects of empagliflozin in preventing PCI-related myocardial injury and this study may be base for direction of future large studies. This study may also have some limitations, such as; small sample size, single center, and short study duration. We also couldn’t measure SYNTAX score in both groups.

## Conclusion

 The present study couldn’t support the potential benefit of empagliflozin on cTnI and hs-CRP levels following elective PCI. Further studies are recommended to show the effects of empagliflozin in prevention of myocardial injury following elective PCI.

## Acknowledgments

 The authors thank the personal of Cardiovascular Research Center of Shahid Madani Heart Center of Tabriz University of Medical Sciences for support of the study.

## Competing Interests

 The authors declare that they have no conflict of interest.

## Ethical Approval

 This study received approval from the Ethics Committee of Tabriz University of Medical Sciences with the ethics code IR.TBZMED.REC.1401.673. Furthermore, the current research was registered at the Iranian Registry of Clinical Trials with the code IRCT20221102056378N1.
